# Appropriate clinical use of darunavir 800 mg

**DOI:** 10.4102/sajhivmed.v19i1.918

**Published:** 2018-10-18

**Authors:** Michelle A. Moorhouse, Sergio Carmona, Natasha Davies, Sipho Dlamini, Cloete van Vuuren, Thandekile Manzini, Moeketsi Mathe, Yunus Moosa, Jennifer Nash, Jeremy Nel, Yoliswa Pakade, Joana Woods, Gert van Zyl, Francesca Conradie, Francois Venter, Graeme Meintjes

**Affiliations:** 1 Wits Reproductive Health and HIV Institute, Faculty of Health Sciences, University of the Witwatersrand, South Africa; 2 National Health Laboratory Services, South Africa; 3 Department of Medicine, University of Cape Town, South Africa; 4 Southern African HIV Clinicians Society, South Africa; 5 Private Practice, Vereeniging, South Africa; 6 Department of Infectious Diseases, University of KwaZulu-Natal, South Africa; 7 Department of Medicine and Institute of Infectious Disease and Molecular Medicine, University of Cape Town, South Africa

## Indication

Darunavir 400 mg tablets were recently approved by the South African Health Products Regulatory Authority (SAHPRA) for the following indication:

PREZISTA, in combination with low dose ritonavir (DRV/r) and with other antiretroviral medicines, is indicated for the treatment of human immunodeficiency virus (HIV) infection in antiretroviral treatment experienced adult patients who are protease-inhibitor-naïve or after exclusion of darunavir resistance associated mutations (DRV-RAMs: V11I, V32I, L33F, I47V, I50V, I54M, I54L, T74P, L76V, I84V and L89V). Genotypic or phenotypic testing should guide the use of DRV/r. (Prezista package insert)

There is no information on the use of darunavir in combination with ritonavir in the paediatric population for the once-daily dose.

## Southern African HIV Clinicians Society guidelines

Southern African HIV Clinicians Society adult antiretroviral therapy (ART) guidelines currently recommend ritonavir-boosted atazanavir (ATV/r) 300/100 mg as preferred boosted protease inhibitor (PI/r) for second-line ART. It was noted in the guidelines that once a suitable tablet for DRV/r 800/100 mg dosing became available, DRV/r 800/100 mg would be a feasible option in second-line ART, with fewer side effects than the DRV/r 600/100 mg twice-daily dosing.

## Using darunavir/ritonavir 800/100 mg once-daily in clinical practice

### In second-line antiretroviral therapy

**FIGURE 1:**
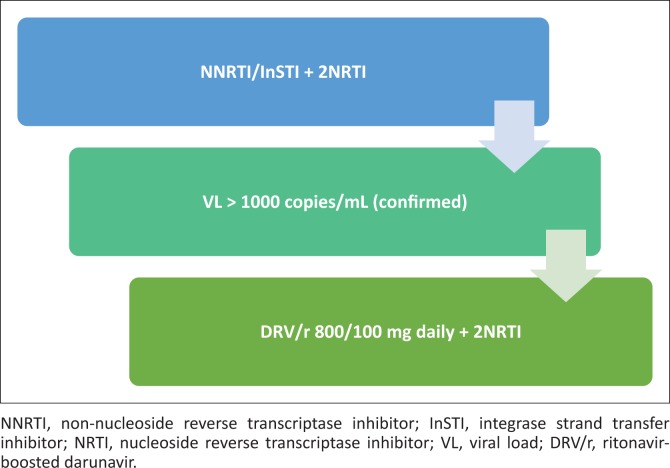
Patients failing non-nucleoside reverse transcriptase inhibitor- or integrase inhibitor-based first-line antiretroviral therapy.

In patients failing first-line non-nucleoside reverse transcriptase inhibitor (NNRTI)- or integrase strand transfer inhibitor (InSTI)-based regimens, switch to DRV/r 800/100 mg daily with two nucleoside reverse transcriptase inhibitors (NRTIs). Sequence the NRTIs as per guidelines (see Figure 1).

**FIGURE 2:**
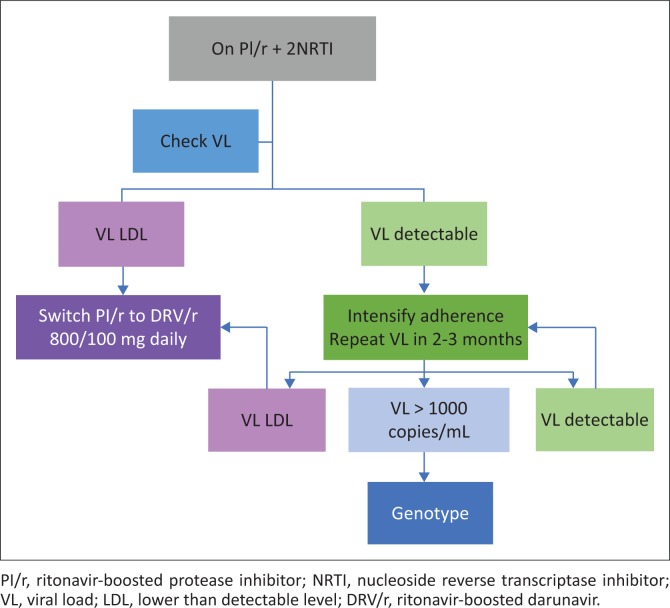
Patients on protease inhibitor-based second-line antiretroviral therapy.

For those patients who are already on a second-line PI/r-based regimen, check the viral load (VL). If the VL is undetectable, then PI/r can be switched to DRV/r 800/100 mg daily, retaining the same NRTI backbone (see Figure 2).

If the VL is detectable, intensify adherence interventions and repeat the VL in 2–3 months. If the VL is undetectable, the PI/r can then be switched to DRV/r 800/100 mg daily. If VL > 1000 copies/mL, resistance genotype is needed to determine if the patient is eligible for third-line ART (see Figure 2).

### Using darunavir/ritonavir 800/100 mg in third-line antiretroviral therapy

Currently, patients on DRV/r on third-line ART receive DRV/r 600/100 mg bid. However, a small proportion of third-line patients have no DRV resistance-associated mutations (RAMs), and in such patients it may be possible to use DRV/r 800/100 mg daily instead of DRV/r 600/100 mg bid to reduce pill burden, dosing frequency and side effects.

**FIGURE 3:**
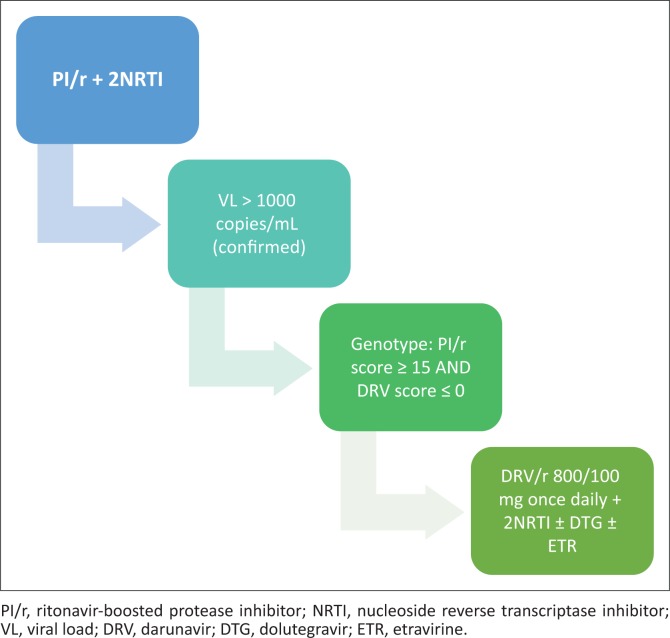
Patients initiating third-line antiretroviral therapy.

For patients initiating third-line ART, if the composite DRV score (Stanford) is zero on *all* genotypes, DRV/r 800/100 mg daily may be initiated (see Figure 3).

**FIGURE 4:**
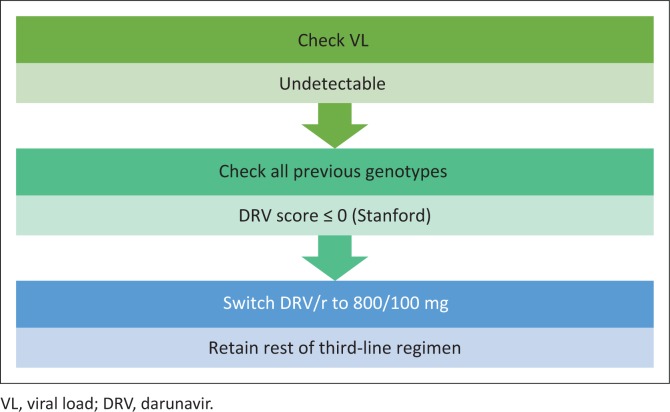
Third-line patients on darunavir/ritonavir-based third-line antiretroviral therapy (600/100 mg bid).

For those patients who are already on a third-line regimen, their VL must be checked. If the VL is undetectable, and the composite DRV score (Stanford) on *all* genotypes is zero, the patient may switch from DRV/r 600/100 mg twice daily to DRV/r 800/100 mg once daily. The rest of the regimen should not be changed (see Figure 4). If the VL is detectable, manage further as appropriate according to current guidelines.

